# Alterations of Hepcidin and Iron Markers Associated with Obesity and Obesity-related Diabetes in Gambian Women

**DOI:** 10.12688/wellcomeopenres.22997.1

**Published:** 2024-11-08

**Authors:** Meike Siemonsma, Carla Cerami, Bakary Darboe, Hans Verhoef, Andrew M. Prentice, Modou Jobe

**Affiliations:** 1 Division of Human Nutrition and Health, Wageningen University & Research, Wageningen, Gelderland, 6700 AA, The Netherlands; 2 Nutrition and Planetary Health Theme, Medical Research Council Unit The Gambia at London School of Hygiene and Tropical Medicine, Fajara, PO Box 273, The Gambia

**Keywords:** Type 2 Diabetes, Obesity, Inflammation, Hepcidin, Iron Metabolism

## Abstract

**Aims:**

Obesity, type 2 diabetes (T2D), and chronic inflammation are associated with disturbances in iron metabolism. Hepcidin is hypothesized to play a role in these alterations owing to its strong association with inflammation via the JAK-STAT3 pathway. The current study investigated the differences between inflammatory markers and iron indices and their association with hepcidin in lean women, women with obesity, and women with obesity and T2D (obesity-T2D) in The Gambia.

**Materials and methods:**

In a cross-sectional study design, fasted blood samples were collected from three groups of women: lean women (n=42, body mass index (BMI)=20.9 kg/m ^2^), women with obesity (n=48, BMI=33.1 kg/m ^2^) and women with obesity-T2D (n=30, BMI=34.5 kg/m ^2^). Markers of inflammation (IL-6 and CRP) and iron metabolism [hepcidin, iron, ferritin, soluble transferrin receptor (sTfR), transferrin, transferrin saturation, and unsaturated iron-binding capacity (UIBC)] were compared using linear regression models. Simple regression analyses were performed to assess the association between hepcidin levels and respective markers.

**Results:**

Women with obesity and obesity-T2D showed elevated levels of inflammatory markers. There was no evidence that markers of iron metabolism differed between lean women and obese women, but women with obesity-T2D had higher transferrin saturation, higher serum iron concentration, and lower UIBC. Serum hepcidin concentrations were similar in all the groups. Hepcidin was not associated with markers of inflammation but was strongly associated with all other iron indices (all P<0.002).

**Conclusion:**

Contrary to our original hypothesis, hepcidin was not associated with markers of inflammation in the three groups of Gambian women, despite the presence of chronic inflammation in women with obesity and obesity-T2D.

## PLAIN LANGUAGE SUMMARY

**What is already known on this topic:**

Obesity, type 2 diabetes (T2D), and chronic inflammation are linked to disturbances in iron metabolism, with hepcidin possibly playing a key role in this relationship. While the role of hepcidin in iron metabolism has been well studied in the context of infectious diseases, its role in metabolic disorders, such as obesity and T2D, remains insufficiently explored.

**What this study adds:**

This study provides new insights into the link between hepcidin, iron indices, and inflammation markers in lean women, women with obesity, and women with obesity-T2D from The Gambia. Surprisingly, no association was found between hepcidin levels and markers of inflammation in women with obesity and obesity-T2D, which contradicts what was initially expected.

**How this study might affect research, practice, or policy:**

This study generated hypotheses regarding the interconnectedness of iron metabolism, insulin metabolism, and pathogenesis of T2D, which should be investigated further in this specific study population. Understanding these mechanisms and links may lead to better care practices and interventions for women with T2D in The Gambia and beyond.

## Introduction

Chronic inflammation, obesity, and type 2 diabetes (T2D) are strongly linked ^1, 2^. Chronic low-grade adipose tissue inflammation in obese patients induces a cascade of processes in the immune system that can result in insulin resistance, hyperinsulinemia, dysregulated glucose homeostasis, β-cell exhaustion, and dysfunction, ultimately leading to T2D ^3^. While the exact pathways of the pathogenesis of T2D are still under investigation, elevated iron stores have been speculated to mediate the development and progression of diabetes and its complications ^4, 5^. This is most likely due to oxidative stress in pancreatic β-cells, which reduces their insulin secretory capacity, resulting in β-cell failure ^6– 9^. Other studies have shown that iron overload can lead to defective insulin binding to receptors and defective insulin clearance, resulting in insulin resistance, as confirmed in animal models ^6^. Interestingly, there appears to be a bidirectional relationship between iron metabolism and diabetes, indicating that alterations in insulin metabolism also affect iron metabolism ^7^. Changes in iron metabolism are observed in patients with diabetes, as well as in obese patients ^10– 13^, with hepcidin potentially central to this relationship ^14^.

**Figure 1.**
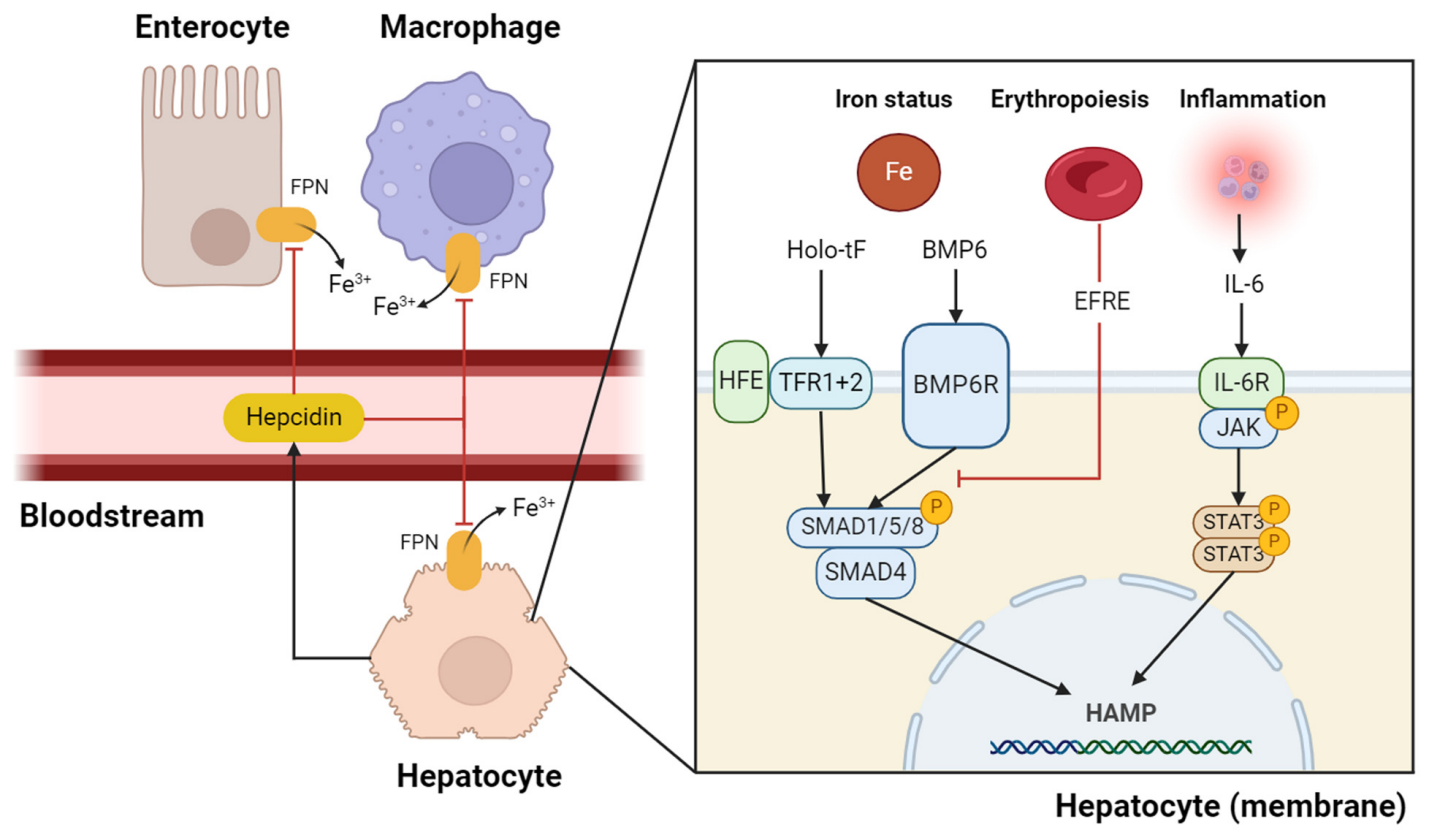
Schematic overview of regulation of hepcidin synthesis. The three major pathways of hepcidin synthesis are iron status, erythropoietic signalling, and inflammatory signalling ^14^. The iron status was measured via HFE, TFR1, TRF2, and BMP6R in the hepatocyte membrane ^14, 16^. When signals of high hepatic stores of iron or high serum iron concentrations are detected, the BMP/SMAD pathway is activated. In a state of inflammation, serum IL-6 activates IL-6R, which in turn activates the JAK/STAT3 pathway ^20^. Both the pathways for detecting iron status and inflammation result in hepcidin mRNA transcription. EFRE, a hormone released by erythroblasts, inhibits hepcidin transcription through mechanisms that are not yet fully understood. However, it is likely to attenuate BMP/SMAD signalling. When hepcidin is released into the bloodstream, it signals to effector cells, such as macrophages and enterocytes, to degrade FPN, so that no more iron is released into the bloodstream from macrophages and less dietary iron is absorbed via the enterocytes. BMP6, bone morphogenetic protein 6, BMP6R= BMP6 receptor; EFRE, erythroferrone; FPN, ferroportin; HAMP, hepcidin antimicrobial peptide; holo-tF, holo-transferrin; HFE, hemochromatosis protein, IL-6R=IL6 receptor; TRF, = transferrin receptor, STAT 3 = signal transducer and activator transcription 3; JAK, Janus kinase. SMAD/1/5/8 should read SMAD 1, 5 or 8. Created using Biorender.com.

The peptide hormone hepcidin is the primary mediator of iron distribution in the body. Hepcidin regulates serum iron by trapping iron in liver cells and macrophages and regulates the absorption of iron in the small intestine ( Figure 1) ^14, 15^. Hepcidin is regulated via three main pathways: iron status, erythropoietic drive, and inflammatory signaling ^16^. The role of hepcidin has been predominantly studied in viral, bacterial, and protozoal infections ^17^. In the case of an infection, inflammation triggers the expression of liver hepcidin, and via this pathway, iron metabolism is closely linked to systematic inflammatory responses in the body ^16– 19^. Hepcidin is also expected to be elevated during low-grade inflammation in patients with chronic diseases such as obesity and/or T2D.

Higher concentrations of interleukin (IL)-6, commonly found in patients with obesity and/or T2D trigger the expression of liver hepcidin via the JAK-STAT3 pathway ^14, 20– 22^. This is believed to be one of the main reasons why obesity is associated with reduced iron absorption, reduced iron stores, and hypoferremia ^13, 21, 23^. The association of T2D with iron status and hepcidin is less clear, with both anemia and iron overload being detected in T2D patients ^24– 27^. Visualization of all the described pathways is shown in *supplemental Figure S1*.

To study the degree to which changes in iron metabolism in obesity and T2D can be linked to alterations in hepcidin levels and chronic inflammation, we studied three groups of Gambian women. In lean women, obese women, and women with obesity-T2D we assessed a) group differences in inflammatory and iron markers, b) associations between hepcidin and markers of inflammation, and c) associations between hepcidin and markers of iron status.

## Methods

### Participants

The participants were originally recruited for Metabolic Endotoxemia and Diabetes in the obese Urban woMen (MEDiUM) study in 2016. Participants were selected using a convenience sampling approach. Sample size calculation was performed for the primary analysis, and the details of this study have been published elsewhere ^28^. This cross-sectional study included three groups of Gambian women (age-range 25–60 years): lean women (BMI: <25.0 kg/m ^2^), women with obesity (BMI ≥30 kg/m ^2^), and women with obesity-T2D (BMI: ≥30 kg/m ^2 ^with confirmed diagnosis of T2D). The women with obesity-T2D were recruited from the Diabetes Clinic at the Edward Francis Small Teaching Hospital in Banjul. The other two groups were recruited via house-to-house screening in Bakau and Serrekunda communities in Kanifing Municipality. Lean women and their obese peers were screened for T2D, and those with a fasting or random capillary glucose concentration of ≥7.0 mmol/L or ≥11.0 mmol/L respectively, were excluded and referred for investigation and management. Participants who were on renal dialysis, had a confirmed diagnosis of inflammatory bowel disease, a self-reported 2-week history of acute febrile illness, a self-reported 2-week history of diarrhea, current use of laxatives or antibiotics, or a 2-week self-reported history of using medication for hyperlipidemia were excluded ^28^. The study details were explained to the participants prior to their consent and recruitment. For the present study, 120 participants were included who completed the study and had their blood collected: lean women (n=42), women with obesity (n=48), women with obesity-related T2D (n=30).

We collected sociodemographic data, household size (those eating from the same cooking pot), level of educational attainment, and household assets (as indicators of socioeconomic status), including the availability of water at home (availability of piped water in household dwellings, piped water into compounds, or open wells in the compound). We also collected anthropometric data (weight, height, and body composition using bioimpedance) and measured the blood pressure. Fasted blood samples were collected after an overnight fast, processed, and frozen at -80°C prior to analysis.

The study protocol was reviewed by the Scientific Coordinating Committee of the MRC Unit, The Gambia London School of Hygiene and Tropical Medicine, and full ethical approval was granted by the Joint Gambia Government/MRC Ethics Committee (SCC 1407).

### Patient involvement

Patients and the general public were not directly involved in the design, conduct, or implementation of this study. This technical approach requires limited direct involvement.

### Anthropometry and body fat assessment

Participants’ height was assessed using a calibrated stadiometer (Leicester height measure, Seca 214, UK) to the nearest 0.1 cm. Body weight (±0.1 kg using digital scales calibrated daily (Tanita BC-418MA, Tanita Corporation, Tokyo, Japan). Other measures of body composition were measured using a segmental analyzer via bioelectrical impedance analysis (Tanita BC-418MA, Tanita Corporation, Tokyo, Japan). Blood pressure was measured three times at one time point using an automated Omron 705IT device. More details on data collection are described elsewhere ^28^.

### Laboratory analysis

Plasma hepcidin concentration was measured using a commercially available ELISA kit (DRG Instruments, Marburg, Germany, cat no: (EIA-5782), according to the manufacturer’s instructions. The measurements were adjusted for the kit used with previously documented regression formulas formulated by Van der Vorm *et al.* to standardize the test results ^29^. Serum iron *(cat no: 03 183 696 122)*, ferritin (cat no: 03 528 995 190), transferrin (cat no: 03 015 050 122), soluble transferrin receptor (sTfR) (cat no: 20 763 454 122), unsaturated iron-binding capacity (UIBC) (cat no: 04 536 355 190), and C-reactive protein (CRP) (cat no: 20 764 930 322) levels were assayed using a Cobas Bio400 biochemical analyzer (Cobas Fara, Roche, UK). IL-6 was measured using a Thermo Fisher Scientific (Life Technologies) IL-6 high-sensitivity ELISA (cat no: BMS213HS, Thermo Fisher Scientific, Waltham, Massachusetts, USA) with a detection limit of 0.03 ng/L. All laboratory analyses were conducted strictly in accordance with the manufacturer’s instructions, which included precise measurements of all reagents specified for the tests.

Transferrin saturation was calculated using the following formula: (total serum iron / [UIBC + total serum iron]) × 100%, with both variables expressed in µmol/L ^30^. The STfR index was calculated using the following formula: (sTfR/Log _10_(ferritin)) ^31^, where sTfR and ferritin were expressed in mg/L and µg/L, respectively.

### Statistical analysis

All analyses were performed using StataBE 17 software (StataCorp, College Station, TX, USA). Population characteristics such as age, cardiometabolic parameters, and demographics are presented for each study group (mean ± SD, unless mentioned otherwise). Iron and inflammation markers were compared using linear regression adjusted for age, which was considered a confounder. Ferritin levels were also adjusted for inflammation (CRP) ( *supplemental Figure S2*). The outcome variables were log-transformed when the distribution of residuals was skewed (serum iron, ferritin, sTfR, CRP, IL-6, UIBC, transferrin saturation). For the models with UIBC and sTfR as an outcome, one outlier in each of the models was removed (UIBC=1.2µmol/L and sTfR=26.6 mg/L) that was >300% of the IQR when the outcome was log-transformed. When log transformation was unsuccessful in normalizing the residuals, a gamma distribution was used to fit the model. This was performed for the model with ferritin as the dependent variable. P-values for group differences are reported and p<0.05 was considered to be strong evidence. The sTfR index was not considered in further analysis because of its highly skewed distribution. To study predictors of hepcidin, univariate analyses were performed using linear regression models with hepcidin as the outcome variable and one of the (transformed) iron/inflammatory markers as the independent variable for all participants grouped together. Sensitivity analysis was performed to determine whether the associations were different for each study group. Interactions by group in each univariate analysis were assessed by comparing multiple fractional polynomial models with and without an interaction (product) term. Finally, a multiple fractional polynomial model was built to determine the covariates (and functions) to be included in the multivariate model predicting hepcidin levels (the mfp procedure was used in Stata, with an acceptance threshold of p < 0.05).

## Results

### Baseline characteristics

The baseline characteristics of the three study groups are shown in Table 1. A total of 120 women were included in this study (flowchart shown in *supplemental Figure S3*). Compared with their lean counterparts, obese women were more educated. Women with obesity-T2D seemed to come from a higher socioeconomic class compared to the two other groups, as indicated by the presence of water sources and flush toilets in the households. As expected, women with obesity and obesity-T2D had a higher fat percentage, higher fat mass index, and lower fat-free mass index than their lean counterparts. These parameters for body composition were similar in obesity and obesity-T2D. T2D was also associated with older age. T2D was also associated with higher fasting blood glucose (FBG) levels, despite all patients being on treatment. Women in the lean and obese groups had FBG levels within the healthy range. Blood pressure levels were highest in women with obesity-T2D and lowest in the lean group. Among the women with obesity-T2D, 57.7% had a transferrin saturation higher than 45%.

**Table 1.** Population characteristics of the study populations (lean women, women with obesity and women with obesity-related T2D).

Variables	Lean women (n=42)	Women with obesity (n=48)	Women with obesity-related T2D (n=30)	Overall p
Sociodemographic parameters				
Age (years) ^†^	37.3±1.2	38.0±1.2	49.2±1.2	p<0.001 ^‡^
Education, n (%)				p=0.68 ^§^
None	22 (52.4)	19 (39.6)	11 (36.7)	
Lower basic (year 1–6)	6 (14.3)	11 (22.9)	8 (30.0)	
Junior secondary (year 7–9)	7 (16.7)	7 (14.6)	4 (13.3)	
Senior secondary and above	7 (16.7)	11 (24.9)	6 (20.0)	
Household size (persons) ^†^	10±1.9	11±1.7	9±1.8	p=0.40 ^‡^
Water in home $, n (%)	34 (80.0)	47 (97.9)	24 (80.0)	p=0.01 ^§^
Flush toilet, n (%)	17 (40.5)	26 (54.2)	26 (86.7)	p<0.001 ^§^
Anthropometric parameters				
BMI (kg/m ^2^) ^†^	20.9±1.1	33.1±1.1	34.5±1.1	p<0.001 ^‡^
Body fat (%)	29.5±5.8	44.4±3.9	46.2±4.1	p<0.001 ^¶^
Fat Mass index (kg/m) ^†^	9.8±1.3	23.7±1.2	25.7±1.2	p<0.001 ^‡^
Fat Free Mass index (kg/m)	23.9±1.4	29.7±2.3	30.1±2.7	p<0.001 ^¶^
Cardiometabolic indicators				
Fasted blood glucose (mmol/L) ^†^	4.8±1.1	5.3±1.1	8.9±1.4	p<0.001 ^‡^
Systolic BP (mmHg) ^†^	117±1.1	128±1.2	144±1.2	p<0.001 ^‡^
Diastolic BP (mmHg) ^†^	74±1.1	79±1.2	86±1.2	p<0.001 ^‡^
Pulse pressure (mmHg) ^†^	42±1.2	48±1.3	57±1.3	p<0.001 ^‡^
Pulse rate (bpm) ^†^	74±1.1	71±1.1	82±1.1	p<0.001 ^‡^
Hypertension, n (%) ^††^	6 (14.3)	13 (27.1)	14 (46.7)	p=0.01 ^§^
Serum inflammatory markers ^‡‡^				
CRP (mg/L) ^†^	1.1±0.9	4.0±2.6	7.1±3.0	p<0.001 ^‡^
IL-6 (ng/L) ^†^	0.9±1.9	1.4±1.9	1.7±1.6	p<0.001 ^‡^
Serum iron markers ^‡‡^				
Hepcidin (µg/L)	7.1±3.5	7.3±3.9	10.5±5.6	p<0.001 ^¶^
Iron (µmol/L)	15.3±1.4	14.6±1.3	17.9±1.3	p=0.02 ^‡^
Ferritin (µg/L) ^†††^	40.1 [18.9-59.4]	35.1 [20.5-57.2]	88.8 [51.1-168.8]	p<0.001 ^¶¶^
Soluble transferrin receptor (sTfR) (mg/L) ^†^	4.4±1.4	4.2±1.4	4.1±1.5	p=0.76 ^‡^
Transferrin (g/L)	3.0±0.5	3.0±0.5	3.0±0.4	p=0.99 ^¶^
Transferrin saturation (%) ^†^	26.8±1.7	24.1±1.6	43.7±1.3	p<0.001 ^‡^
Transferrin saturation > 45%, n (%) ^§§^	8 (19.1)	5 (10.4)	17 (56.7)	p<0.001 ^¶^
UIBC (µmol/L) ^†^	26.0±1.6	24.1±1.6	43.7±1.6	p<0.001 ^‡^
sTfR index ^‡‡‡, †††^	2.6 [2.2-4.2]	2.5 [2.0-3.7]	1.9 [1.7-2.6]	p=0.04 ^¶¶^

T2D: Type-2 diabetes, BMI, body mass index; CRP, C-reactive protein; UIBC, unsaturated iron-binding capacity. ^†^GM±SD: geometric mean ± geometric standard deviation (SD ^‡^ANOVA test with log-transformed variables to meet the assumption of a normal distribution ^§^Fisher’s exact test ^¶^ANOVA test ^††^Based on cut-off values of SDB ≥ 140 mmHg and/or DBP ≥ 90 mmHg. ^‡‡^5–12 missing data points, varying per variable ^§§^Measures that could indicate iron overload ^32^ ^¶¶^Kruskal-Wallis test (when log transformation was unsuccessful in obtaining a normal distribution). ^†††^Median [IQR] ^‡‡‡^(sTfR/10Log (ferritin))

### Markers of inflammation and iron metabolism

**Figure 2.**
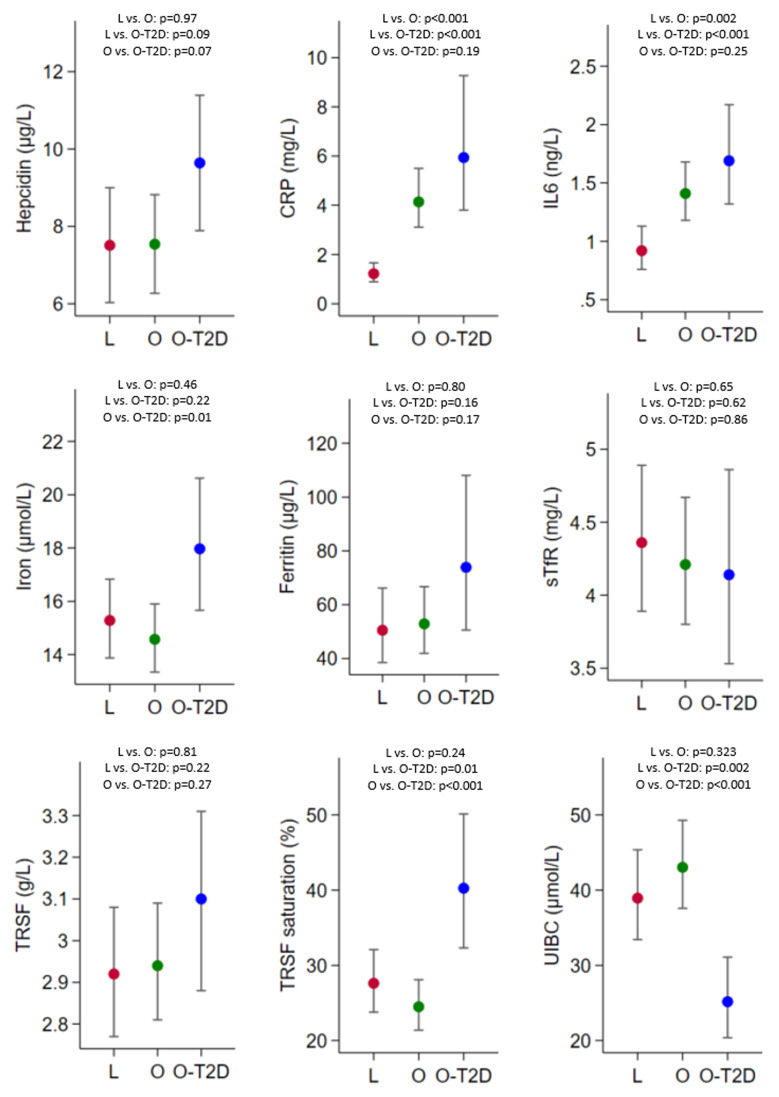
Paired group comparisons of age-adjusted (at mean) predicted means and 95%CIs of markers of inflammation (CRP and IL-6) and markers of iron metabolism (iron, hepcidin, ferritin, sTfR, TRSF, TRSF saturation, UIBC). L, lean women (red); O, obese women (green); O-T2D, obese women with type 2 diabetes (blue). CRP, C-reactive protein; IL-6, interleukin 6; sTfR, soluble transferrin receptor; TRSF, transferrin. In addition to age, ferritin was also adjusted for inflammation, as it changed the measure of association by >10% ( *supplementary Figure 2*). Note that not all the y-axes start at zero.

Figure 2 shows the predicted means and 95% CIs of the markers of inflammation and iron metabolism. We observed lower serum concentrations of CRP and IL-6 in lean women. These values were higher and similar in women with obesity and obesity-T2D. The iron concentration was higher in women with obesity than in women with obesity-T2D. There is some indication that serum concentrations of hepcidin and ferritin are higher in women with obesity-T2D, compared to the other two study groups; however, no strong evidence was found. The serum concentrations of sTfR and transferrin were similar in all groups. Finally, transferrin saturation values were higher and UIBC was lower in women with obesity-T2D compared than in the other two groups, while they were similar in lean women and women with obesity.

### Predictors of hepcidin concentration

**Figure 3.**
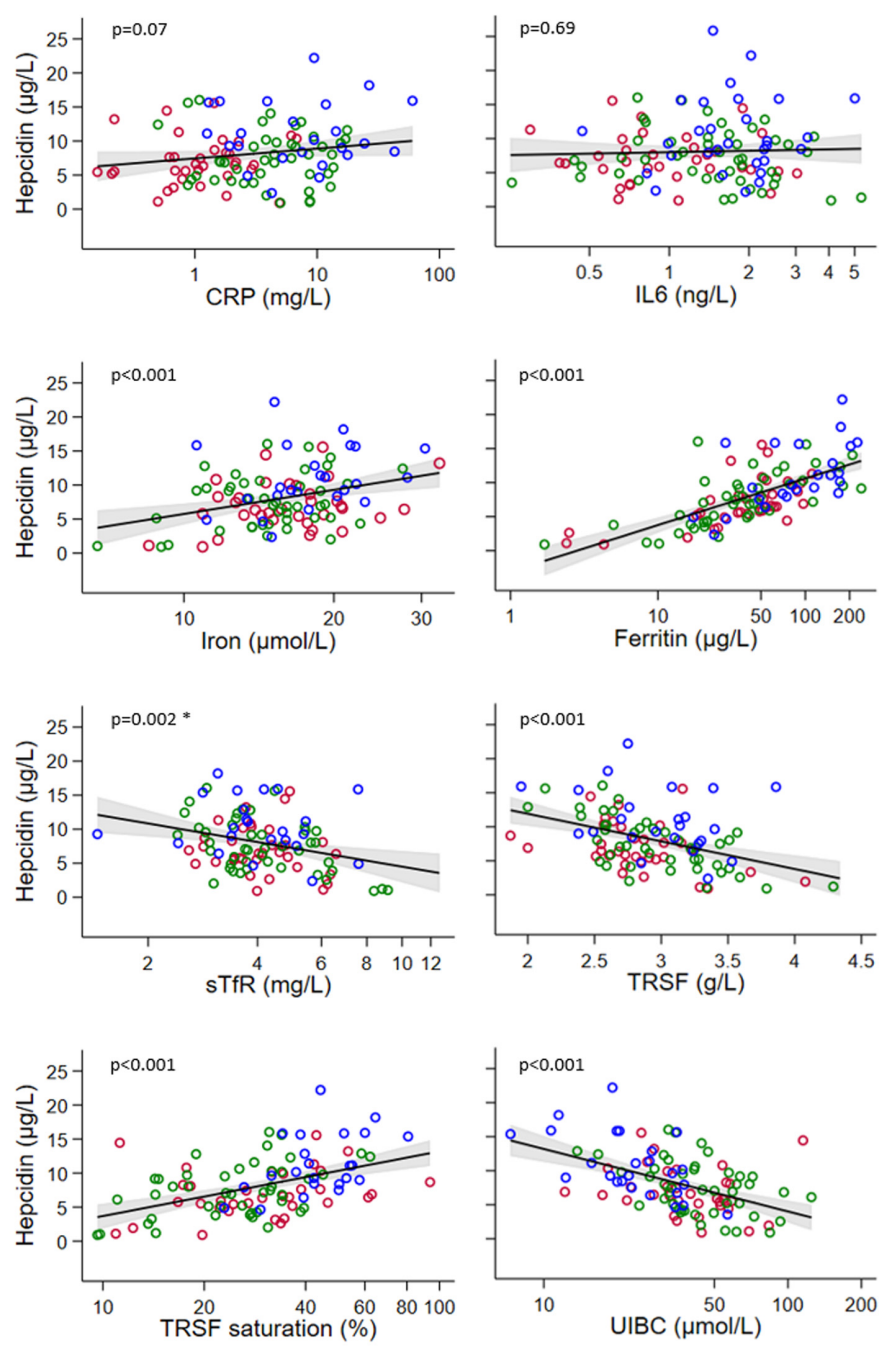
Association of hepcidin with (log transformed) markers of inflammation (CRP and IL-6) and serum iron markers (iron, hepcidin, ferritin, sTfR, TRSF, TRSF saturation, UIBC) for the three study groups grouped together. Red = lean women, green = women with obesity, blue = women with obesity-T2D. CRP: C-reactive protein, IL-6: Interleukin 6, sTfR: soluble transferrin receptor, TRSF=transferrin. The red line in each figure indicates the fitted regression line and the grey area indicates the 95% confidence band for this line. * One influential outlier (7.2, 22.2) was removed ( *supplementary Figure 4*).

Figure 3 shows the association between hepcidin with levels and the markers of inflammation and iron metabolism. The strengths and natures of the associations were similar in each study group. Therefore, the results for the three study groups were grouped to fit the regression models. No association was found between hepcidin and the inflammatory markers, IL-6 and CRP. Strong evidence was found for a positive association between hepcidin and log serum iron, log ferritin, and log transferrin saturation as well as a negative association between hepcidin and log sTfR, transferrin, and log UIBC (all p<0.002). The strongest association was observed between ferritin and hepcidin levels. Age (continuous) was also a predictor of hepcidin serum concentrationlevels (p<0.001). Multivariable fractional polynomial modelling showed that the best model for predicting hepcidin was a univariate model with log-transformed ferritin as the predictor, which was shown to be better than a multivariate model.

## Discussion

We did not observe differences in iron metabolism markers between lean women and obese women, but we found evidence of elevated iron stores in the obese-T2D group. Raised serum hepcidin concentrations were observed in the obesity-T2D group, but no strong evidence was found. Moreover, hepcidin was not associated with the inflammatory markers CRP and IL-6, whereas all other measures of iron markers were strong (dependent) predictors of hepcidin serum concentration.

The elevated levels of IL-6 and CRP in women with obesity and obesity-T2D indicate that these groups indeed suffer from chronic inflammation, which agrees with other studies in both Western populations and people living in sub-Saharan Africa (SSA) ^33, 34^. We hypothesized that chronic inflammation is associated with the upregulation of hepcidin. However, in the current study, all markers of iron metabolism, including hepcidin, were similar in lean women and women with obesity. While it is generally accepted that obesity is associated with reduced iron absorption, lower iron stores, and hypoferremia in Western contexts, different patterns have been observed in African contexts ^23^. Several studies on SSA have not found a link between lower iron stores, micronutrient deficiencies, and obesity ^35– 37^. While a double burden of malnutrition exists in many countries in SSA, evidence for the double burden within the same individual is weak. A study by Williams *et al.*, in which the association between iron status and obesity was studied in 17 countries, the general trend showed that in LMICs, including several African countries, obese and overweight women were less likely to suffer from anemia or micronutrient deficiencies compared to lean individuals ^38^. It is important to consider that in Western countries, obesity is associated with lower socioeconomic status and linked to diets low in micronutrients, while the opposite is true in many African countries, such as The Gambia ^28^. It has been shown that in most countries in SSA, higher SES is associated with higher rates of overweight and obesity, as well as lower rates of anemia ^39^. This could explain the differences in the findings in the Western and African contexts.

Notably, higher levels of transferrin saturation and lower levels of UIBC in women with obesity-T2D could indicate higher body iron stores in these women than in lean women and women with obesity only. The trend of higher serum iron and ferritin levels (unadjusted and adjusted for inflammation) adds to this evidence. A high proportion (57%) of iron overload (transferrin saturation >45%) among women with obesity-T2D is notable. Higher transferrin saturation is associated with higher mortality rates and faster progression of diabetes and its complications, making it a relevant marker for further investigation ^40^. T2D has been linked to elevated levels of transferrin saturation before, especially at a more advanced stage of disease, but research is inconsistent as T2D has also been linked to anemia ^10, 24– 26^. Similarly, both elevated and lower hepcidin levels have been reported in T2D patients ^10^. The two can even occur at the same time as T2D patients who present with iron overload can still have subclinical anemia, indicated by lower hemoglobin concentrations ^10^. The strong association of hepcidin with all other measured iron markers indicates that hepcidin is well regulated via pathways of body iron stores, and is similar to other studies that look into the association of hepcidin and markers of iron metabolism ^41, 42^. One hypothesis is that women with T2D may develop hepcidin resistance, which reduces the ability of hepcidin to lower iron stores.

However, this does not explain why we did not find any association between inflammatory markers and hepcidin serum concentrationlevels in women with obesity-T2D, where we would expect an association. It has been suggested that in chronic inflammation, a certain threshold of inflammation needs to be reached to affect iron metabolism via JAK-STAT3, but also at higher levels of inflammation; no association was found ^35^. An alternative hypothesis is that the JAK-STAT3 pathway, which regulates hepcidin expression via IL-6, is disturbed by another mechanism. Several studies have found evidence of a link between hepcidin and insulin metabolism, which is disturbed in patients with obesity and/or diabetes ^43^. Lower hepcidin levels are linked to insulin resistance, and insulin sensitivity is improved by reducing the iron load via blood donations, indicating a causal relationship ^44– 48^. Interestingly, insulin resistance has been associated with disturbances in the STAT3 pathway and regulation of hepcidin ^49^. The strongest evidence came from a study by Wang *et al.*, in which lower hepcidin levels were observed in streptozotocin-induced diabetic rats ^50^. When insulin was administered, hepcidin expression was upregulated, and hepcidin serum concentrations increased. When the STAT3 pathway is blocked, the effect of insulin on hepcidin disappears, suggesting that insulin directly affects hepcidin regulation via this pathway. This is a plausible hypothesis for the missing association between inflammation and hepcidin in women with obesity (who might already suffer from insulin resistance) and obesity-T2D, as well as elevated levels of iron in the obesity-T2D group, at a more advanced stage of the disease. In lean women, only very low concentrations of inflammatory markers were observed, which were probably not critical for hepcidin expression ^35^. If this hypothesis is correct, treatment with insulin in women with obesity-T2D might not only correct elevated blood glucose levels (poorly controlled in this group) but could also correct hepcidin levels and lower iron stores, which are linked to alleviated insulin resistance ^14^. In this case, the benefit of insulin treatment in this patient group would be two-fold. Further research is required to confirm this hypothesis.

The main strength of this study is that we investigated the role of hepcidin in alterations of iron metabolism in obesity and obesity-T2D in an understudied population in which different patterns arise compared to studies in Western contexts. Even with a small sample size, strong associations between hepcidin and other iron markers were observed. This study has several limitations. First, in this study, we looked at a limited number of iron markers that can provide some indications of iron status but do not provide a complete picture of the iron profile of the participants, such as anemia status. Second, the cross-sectional nature of the study did not allow us to investigate causal relationships, but merely into associations of markers of inflammation and iron metabolism at one time point. Third, no information was collected on dietary intake or insulin metabolism; therefore, we could not test the hypotheses that were generated based on the study results. Finally, the women with obesity-T2D were substantially older than the other two groups, and the observed differences could be partly attributed to the age gap, rather than diabetes and/or obesity, even after adjusting for it. An observational study showed that menopause is associated with higher iron stores ^51^. However, the same study showed a strong association between elevated iron markers and insulin resistance, which again confirms the link between iron metabolism and insulin metabolism.

In conclusion, the current study confirmed that obesity and obesity-T2D are associated with markers of chronic inflammation, but it did not confirm our hypothesis that hepcidin is linked to these markers in three groups of Gambian women, of whom two were suffering from chronic inflammation due to obesity and/or T2D. The interactions of diet and insulin metabolism with hepcidin and iron status should be further investigated, as well as other possible explanations.

## Ethics and consent

### Ethics statement

The study protocol was reviewed by the Scientific Coordinating Committee of the MRC Unit, The Gambia London School of Hygiene and Tropical Medicine, and full ethical approval was granted by the Joint Gambia Government/MRC Ethics Committee on 13 March 2015 (approval number: SCC 1407). The study was conducted in accordance with the ethical principles outlined in the Declaration of Helsinki. (SCC 1407).

### Consent to participate

The study details were explained to the participants prior to their consent and recruitment. Detailed study information was explained to study participants by a trained field assistant prior to obtaining signed or thumb printed consent. The procedure of obtaining written consent from all participants was approved by the Join Gambia Government/MRC Ethics Committee. The ethical approval committee granted a waiver for the full formal consent procedure.

## Data Availability

The protocol approved by the Joint Gambia Government/MRC Ethics Committee does not include sharing data beyond the investigators and requires a data transfer agreement prior to data sharing. The data that support the findings of this study are available upon request from Modou Jobe ( modou.jobe@lshtm.ac.uk) and after a data transfer agreement is in place, as required by local regulations. Open Science Framework: Alterations of Hepcidin and Iron Markers associated with Obesity and Obesity-related Diabetes in Gambian Women. https://doi.org/10.17605/OSF.IO/JPV56. This project contains the following extended data: Data file 1. (Supplementary material study) Data file 2. (STROBE checklist for cross-sectional studies) Data are available under the terms of the Creative Commons Attribution 4.0 International license (CC-BY 4.0). Siemonsma, M. (n.d.). *Alterations of Hepcidin and Iron Markers associated with Obesity and Obesity-related Diabetes in Gambian Women*. Open Science Framework. https://osf.io/jpv56/ doi: 10.17605/OSF.IO/JPV56 ^52^. Data are available under the terms of the Creative Commons Attribution 4.0 International License (CC BY 4.0).

## Software availability

The analyses presented in this study were conducted using Stata, a proprietary software available from StataBE 17 software (StataCorp, College Station, TX, USA). However, the analyses can also be performed using free and open-source software, such as R Statistical Software.
