# Supplementary material for: Alterations of Hepcidin and Iron Markers Associated with Obesity and Obesity-related Diabetes in Gambian Women
Source: Wellcome Open Res. 2025 Sep 9;9:666. Originally published 2024 Nov 8. [Version 2] doi: 10.12688/wellcomeopenres.22997.2 (PMC12398679; doi:10.12688/wellcomeopenres.22997.2)
Supplement: Peer review history

# Reviewer report: Reviewer response for version 2

**DOI:** 10.21956/wellcomeopenres.27483.r132039

**Published:** 2025-09-23

**Authors:** Omar Obeid

**Affiliations:** 1 American University of Beirut, Beirut, Lebanon

no further comments to make

Is the work clearly and accurately presented and does it cite the current literature?

Yes

If applicable, is the statistical analysis and its interpretation appropriate?

Yes

Are all the source data underlying the results available to ensure full reproducibility?

Yes

Is the study design appropriate and is the work technically sound?

Yes

Are the conclusions drawn adequately supported by the results?

Yes

Are sufficient details of methods and analysis provided to allow replication by others?

Yes

Reviewer Expertise:

nutrition, micronutrients, metabolism

I confirm that I have read this submission and believe that I have an appropriate level of expertise to confirm that it is of an acceptable scientific standard.

# Reviewer report: Reviewer response for version 1

**DOI:** 10.21956/wellcomeopenres.25325.r127371

**Published:** 2025-08-30

**Authors:** Nathalie J. Lambrecht

**Affiliations:** 1 Stanford University, Stanford, USA

The study examined the associations between hepcidin levels and markers of inflammation and iron status in a sample of Gambian women. The cross-sectional study included lean women, women with obesity, and women with obesity-associated type 2 diabetes (obesity-T2D). The authors found higher levels of inflammatory markers in women with obesity and obesity-T2D, as well as higher iron in women with obesity-T2D. There were no differences in hepcidin concentrations in the three groups, nor was hepcidin associated with markers of inflammation. Hepcidin was associated with iron markers in the expected directions.

 The study is comprehensive and appropriately explores the relationships between the three groups and biomarkers. The manuscript is well-written and adequately places the study within the context of current literature.

- It is interesting that you find elevated iron stores in women with obesity-T2D, contrary to what would be expected. Your explanation in the discussion makes sense. Could it also be that that the degree of obesity in your study sample is insufficient to cause low iron status? In the study in a Western context that you cite, women with obesity had an average BMI of 38, much higher than in your sample. This is something we have previously hypothesized as well among a sample of Chinese women (Jones et al., 2021; https://doi.org/10.1093/jn/nxab133).
- In the introduction, I think it would be helpful to expand on the line that reads “Changes in iron metabolism are observed in patients with diabetes, as well as in obese patients…”. Providing more context on the expected directionality would help readers who are less familiar with current literature on iron status in these patient groups.
- I could not access the supplemental documents. Please ensure these are readily available to readers.

Minor points:

- In the abstract, materials and methods, clarify that you are providing mean BMI for each of the groups.
- In the Figure 1 caption, should this say, “Iron status is measured via…”?
- There are issues with parentheses in the following lines that should be fixed:
  - “Body weight (±0.1 kg using digital scales calibrated daily (Tanita BC-418MA, Tanita Corporation, Tokyo, Japan).”
  - “Plasma hepcidin concentration was measured using a commercially available ELISA kit (DRG Instruments, Marburg, Germany, cat no: (EIA-5782), according to the manufacturer’s instructions.”
  - Fig 1 footnote: “GM±SD: geometric mean ± geometric standard deviation (SD”
- What does the “$” after water in home in Table 1 signify?
- Rewrite this sentence for clarity: “Figure 3 shows the association between hepcidin with levels and the markers of inflammation and iron metabolism.”
- Provide an abbreviation definition for UIBC in Figure 2 and Figure 3.
- Consider revising the conclusion sentence for clarity and splitting into two sentences: “In conclusion, the current study confirmed that obesity and obesity-T2D are associated with markers of chronic inflammation, but it did not confirm our hypothesis that hepcidin is linked to these markers in three groups of Gambian women, of whom two were suffering from chronic inflammation due to obesity and/or T2D.”

Is the work clearly and accurately presented and does it cite the current literature?

Yes

If applicable, is the statistical analysis and its interpretation appropriate?

Yes

Are all the source data underlying the results available to ensure full reproducibility?

Yes

Is the study design appropriate and is the work technically sound?

Yes

Are the conclusions drawn adequately supported by the results?

Yes

Are sufficient details of methods and analysis provided to allow replication by others?

Yes

Reviewer Expertise:

nutrition, global health, food systems

I confirm that I have read this submission and believe that I have an appropriate level of expertise to confirm that it is of an acceptable scientific standard.

# Response

**Published:** 2025-09-05

**Authors:** Meike Siemonsma

**Affiliations:** Division of Human Nutrition and Health, Wageningen University & Research, Wageningen, Gelderland, The Netherlands

We would like to sincerely thank the reviewer for their constructive feedback on our manuscript. We are grateful for their positive assessment of our study design, writing, and contextualization within the current literature. Below, we address each of the reviewer’s comments in turn.

**Comment:** *It is interesting that you find elevated iron stores in women with obesity-T2D, contrary to what would be expected. Your explanation in the discussion makes sense. Could it also be that the degree of obesity in your study sample is insufficient to cause low iron status? In the study in a Western context that you cite, women with obesity had an average BMI of 38, much higher than in your sample. This is something we have previously hypothesized as well among a sample of Chinese women (Jones et al., 2021).*

**Response:** We thank the reviewer for this suggestion. We agree that the degree of obesity and associated inflammation may not always be sufficient to induce the lower iron status typically observed in populations with more severe obesity. We examined our data for associations between hepcidin and inflammatory markers in participants with the highest BMI and inflammation levels, but found no indication of this relationship in the more severe cases, as described in the discussion. While the reviewer's explanation regarding obesity severity, as suggested by Jones et al. (2021) in pregnant Chinese women, could be valid in certain contexts, our data do not support this as the primary explanation for our findings. The absence of expected associations even among our participants with the highest obesity and inflammation levels suggests that factors beyond obesity severity may be at play in this population. We have incorporated the suggested reference (Jones et al., 2021) to provide broader context for interpreting these findings and acknowledge this alternative explanation in our discussion.

**Comment:** *In the introduction, expand on the line that reads “Changes in iron metabolism are observed in patients with diabetes, as well as in obese patients…” Providing more context on the expected directionality would help readers less familiar with the literature.*

**Response:** We appreciate this suggestion and have expanded this sentence in the Introduction to clarify the expected directionality of changes in iron metabolism in both obesity and diabetes, with reference to relevant literature. Note that we discuss the direction further in the next section. However, immediately mentioning this in the first sentence will help readers understand the rest of the text.

**Comment:** *I could not access the supplemental documents. Please ensure these are readily available to readers.*

**Response:** We apologize for this. We have changed the settings of the link with the supplemental documents to ensure they are accessible to all readers.

**Minor points:**

1. *In the abstract, materials and methods, clarify that you are providing mean BMI for each of the groups.*
**Response:** This has been clarified in the abstract (geometric mean was used).
2. *In the Figure 1 caption, should this say, “Iron status is measured via…”?*
**Response:** Thank you for pointing this out. The caption has been revised.
3. *There are issues with parentheses in the following lines…*
**Response:** We have corrected the formatting of the parentheses in the Methods section and Figure 1 footnote.
4. *What does the “$” after water in home in Table 1 signify?*
**Response:** This was a typographical error and has been removed. We have explained in the methods what “water in home” indicates. Earlier this was done in the table itself, where we forgot to remove the dollar sign.
5. *Rewrite this sentence for clarity: “Figure 3 shows the association between hepcidin with levels and the markers of inflammation and iron metabolism.”*
**Response:** This sentence has been revised to: *“Figure 3 shows the association between hepcidin levels and markers of inflammation and iron metabolism.”*
6. *Provide an abbreviation definition for UIBC in Figure 2 and Figure 3.*
**Response:** We have added the definition of UIBC (Unsaturated Iron Binding Capacity) to the figure legends.
7. *Consider revising the conclusion sentence for clarity and splitting into two sentences.*
**Response:** The conclusion has been revised for clarity and is now presented in two sentences as suggested.

We thank the reviewer once again for their careful review and constructive feedback, which have strengthened the clarity and impact of our manuscript.

# Reviewer report: Reviewer response for version 1

**DOI:** 10.21956/wellcomeopenres.25325.r115677

**Published:** 2025-01-23

**Authors:** Omar Obeid

**Affiliations:** 1 American University of Beirut, Beirut, Lebanon

- The manuscript is well written, and results are presented in the appropriate forms.
- Discussion is very clear.
- The failure to find a correlation between hepcidin and inflammatory markers was adequately discussed. 
- Minor input is needed on the difference in age between the groups, especially since all significances appeared in subjects with both obesity and T2D, which were of an older age. in the text, age was mentioned to be a good predictor of hepcidin. 

Is the work clearly and accurately presented and does it cite the current literature?

Yes

If applicable, is the statistical analysis and its interpretation appropriate?

Yes

Are all the source data underlying the results available to ensure full reproducibility?

Yes

Is the study design appropriate and is the work technically sound?

Yes

Are the conclusions drawn adequately supported by the results?

Yes

Are sufficient details of methods and analysis provided to allow replication by others?

Yes

Reviewer Expertise:

nutrition, micronutrients, metabolism

I confirm that I have read this submission and believe that I have an appropriate level of expertise to confirm that it is of an acceptable scientific standard.

# Response

**Published:** 2025-09-04

**Authors:** Meike Siemonsma

**Affiliations:** Division of Human Nutrition and Health, Wageningen University & Research, Wageningen, Gelderland, The Netherlands

We would like to thank the reviewer for their feedback on our manuscript. We also appreciate their positive remarks regarding the clarity of our writing, results presentation, and discussion. 

**Comment:**

 Minor input is needed on the difference in age between the groups, especially since all significances appeared in subjects with both obesity and T2D, which were of an older age. In the text, age was mentioned to be a good predictor of hepcidin. 

**Response:**

 We appreciate the reviewer highlighting the role of age in our analysis. As noted in the manuscript, we have already adjusted for age in our statistical models to account for its potential confounding effect on iron and inflammation markers. While we acknowledge that some residual confounding by age may still be present, further adjustment is not possible within the current analytical framework. However, to address this point, we have added additional commentary in the Discussion section to explicitly highlight the age differences between groups and their potential influence on our findings. Thank you once again for your time and constructive feedback.
